# The Association of Platelet Count with Clinicopathological Significance and Prognosis in Renal Cell Carcinoma: A Systematic Review and Meta-Analysis

**DOI:** 10.1371/journal.pone.0125538

**Published:** 2015-05-08

**Authors:** Liangyou Gu, Hongzhao Li, Yu Gao, Xin Ma, Luyao Chen, Xintao Li, Yu Zhang, Yang Fan, Xu Zhang

**Affiliations:** Department of Urology/State Key Laboratory of Kidney Diseases, Chinese PLA General Hospital/PLA Medical School, No. 28 Fuxing Road, Beijing 100853, P.R. China; Southern Illinois University School of Medicine, UNITED STATES

## Abstract

**Objective:**

Elevated platelet count (PC), a measure of systemic inflammatory response, is inconsistently reported to be associated with poor prognosis in patients with renal cell carcinoma (RCC). We conducted a systematic review and meta-analysis to clarify the significance of PC in RCC prognosis.

**Methods:**

PubMed, Embase, and Web of Science databases were searched to identify eligible studies to evaluate the associations of PC with patient survival and clinicopathological features of RCC.

**Results:**

We analyzed 25 studies including 11,458 patients in the meta-analysis and categorized the included articles into three groups based on RCC stage. An elevated PC level was associated with poor overall survival (OS, hazard ratio [HR] 2.24, 95% confidence interval [CI] 1.87-2.67, *P*<0.001) and cancer-specific survival (CSS, HR 2.59, 95% CI 1.92-3.48, *P*<0.001) when all stages were examined together; with poor CSS (HR 5.09, 95% CI 2.41-10.73, *P*<0.001) and recurrence-free survival (HR 6.68, 95% CI 3.35-13.34, *P*<0.001) for localized RCC; with poor OS (HR 2.00, 95% CI 1.75-2.28, *P*<0.001) for metastatic RCC; and with poor OS (HR 2.05, 95% CI 1.04-4.03, *P* = 0.038), CSS (HR 3.38, 95% CI 1.86-6.15, *P*<0.001), and PFS (HR 2.97, 95% CI 1.47-6.00, *P* = 0.002) for clear cell RCC. Furthermore, an elevated PC level was significantly associated with TNM stage (OR 3.11, 95% CI 1.59-6.06, *P* = 0.001), pathological T stage (OR 3.13, 95% CI 2.60-3.77, *P*<0.001), lymph node metastasis (OR 4.01, 95% CI 2.99-5.37, *P*<0.001), distant metastasis (OR 3.85, 95% CI 2.46-6.04, *P*<0.001), Fuhrman grade (OR 3.70, 95% CI 3.00-4.56, *P*<0.001), tumor size (OR 4.69, 95% CI 2.78-7.91, *P*<0.001) and Eastern Cooperative Oncology Group score (OR 5.50, 95% CI 3.26-9.28, *P*<0.001).

**Conclusion:**

An elevated PC level implied poor prognosis in patients with RCC and could serve as a readily available biomarker for managing this disease.

## Introduction

Renal cell carcinoma (RCC), an aggressive malignancy whose incidence of occurrence is rising alarmingly, accounts for 2–3% of cancers in adults, and is the second leading cause of death from urological cancers [1]. Approximately 20–30% of patients with RCC present with metastatic disease at initial diagnosis, and another 30% of patients with apparently localized disease, who undergo curative surgery, will eventually develop metastasis [2]. Hence, a means of identifying patients with poor prognoses, who may benefit from aggressive treatment, is greatly needed. Although post-operative histopathological parameters are currently the most widely used predictors to stratify patients [3], these variables might not be entirely reliable; further improvement in preoperative prognostication is warranted [4]. Therefore, a biological marker, which can enhance outcome prediction, and help identify patients at greater risk, is required to precisely guide clinical decisions.

A systemic inflammatory response has been shown to be a prognostic indicator for several advanced cancers [5,6]. Although an elevated platelet count (PC), a measure of systemic inflammatory response, has been associated with poor RCC prognosis in several studies, variations in results have precluded a consensus. A 2011 meta-analysis found elevated PC to predict poor survival in patients with RCC [7]. However, this study combined all data together with no subgroup analysis, and only looked at overall survival (OS) and cancer-specific survival (CSS). Further, it could not include later studies (which had more reliable data). For an updated analysis and more detailed conclusions, we conducted a systematic review of published studies in order to evaluate the prognostic value of PC by exploring the associations of PC with survival and the clinicopathological features of RCC. If the extracted data could be merged, a meta-analysis was conducted.

## Materials and Methods

### Search strategy and selection criteria

This meta-analysis was conducted in accordance with the guidelines of Preferred Reporting Items for Systematic Reviews and Meta-Analyses (PRISMA) [8]. The PRISMA 2009 checklist for our study is shown in the supplementary materials (S1 PRISMA Checklist).

A literature search for studies published by August 31, 2014 that assessed the effect of platelet count (PC) in RCC was performed in PubMed, Embase, and Web of Science using the following search items through MeSH headings, keywords, and text words: (“renal cancer” or “kidney cancer” or “renal carcinoma” or “renal cell carcinoma”) and (“platelet” or “platelet count” or “thrombocytosis” or “thrombocythemia”). Additionally, we manually screened the references of the relevant literature, including all of the identified studies, reviews, and editorials. The inclusion criteria were as follows: (1) treatment limited to surveillance, surgery, targeted therapy, or immunotherapy; (2) measurement of PC before specific treatments and analysis of its potential association with RCC; (3) retrospective or prospective study design; and (4) median follow-up of at least 6 months. Articles were excluded if they (1) were not written in English; (2) were letters, case reports, meeting records or review articles; (3) sampled fewer than 40 patients; (4) dealt with PC level as a continuous rather than a dichotomized variable; or (5) lacked sufficient data for estimating hazard ratios (HRs) and their 95% confidence intervals (CIs). When duplicate studies were retrieved, we included the more informative and recent article. Three researchers (L.Y.G., H.Z.L., and Y.G.) identified all the studies that fit the inclusion criteria for full review. Discrepancies were resolved through discussion.

### Quality assessment

**Fig 1:**
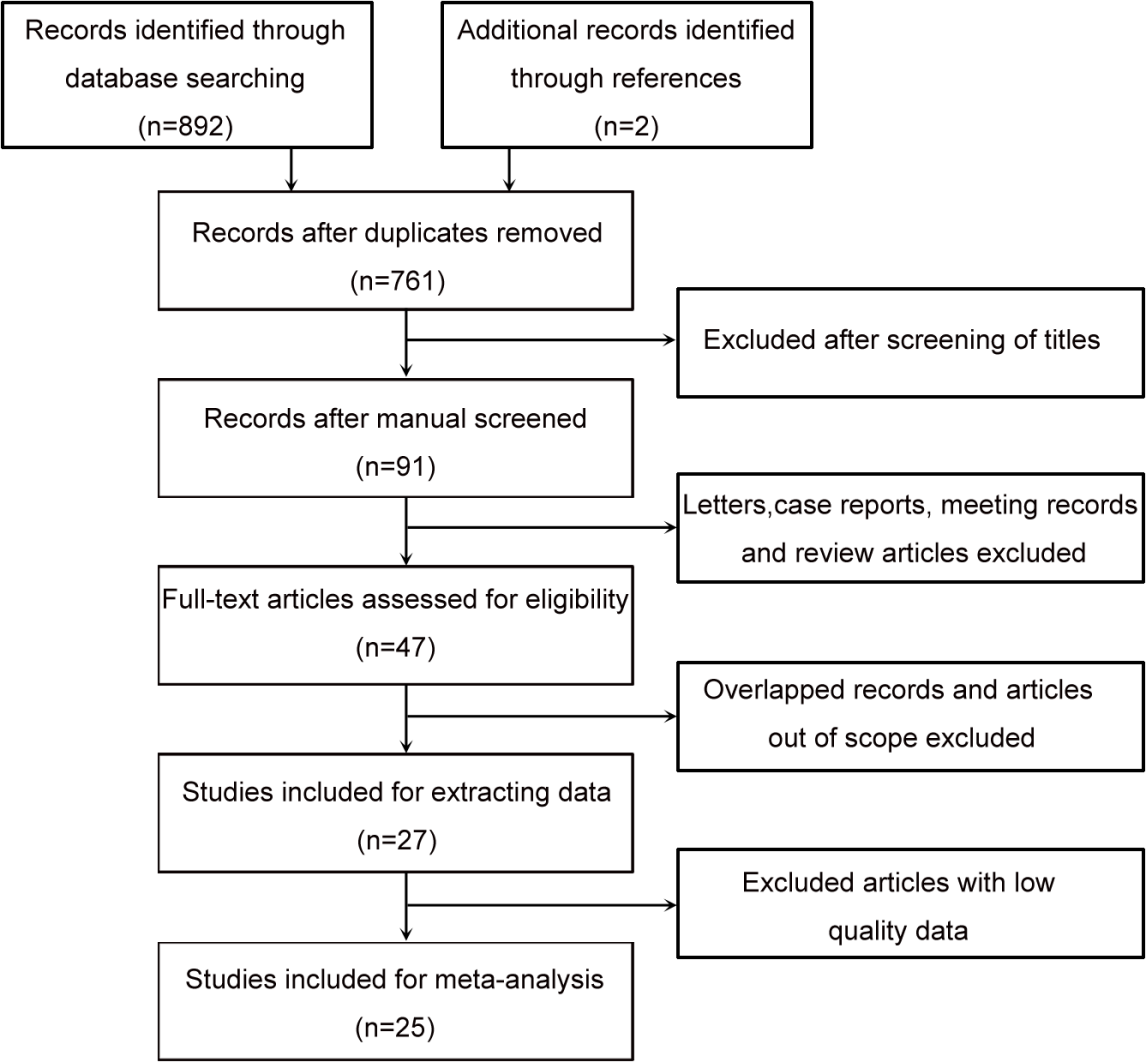
Flowchart of selecting studies for inclusion in this meta-analysis.

In accordance with the guidelines of the meta-analysis of observational studies in epidemiology, we assessed the quality of all the studies included [9]. Key checklist points included (1) clearly defined study design, (2) clearly described study population, (3) sufficiently large sample (*N* > 40 for the current study), (4) clearly described outcome assessment (overall survival [OS], cancer-specific survival [CSS], progression-free survival [PFS], or recurrence-free survival [RFS]), and (5) sufficiently long follow-up. To ensure the quality of this meta-analysis, studies were excluded if they did not meet these five criteria. All selected studies were nonrandomized, and a flowchart of the study selection process is shown in Fig 1.

### Data extraction and conversion

Data were extracted independently by three investigators (L.Y.G., H.Z.L., and Y.G.), who used a predefined form to extract all relevant information, including the first author’s last name, year of publication, study location, number of patients, cut-off value, median and range of patient age, and follow-up period.

Twenty-five studies that focused on the association between RCC and PC were included for meta-analysis. The studies were divided into three groups: the all-stage group, which contained eleven studies of RCC patients at all stages [4,10–19]; the localized group, which contained five studies of patients with localized RCC only [20–24]; and the metastatic group, which contained nine studies of patients with metastatic RCC only [25–33]. The patients in each study were divided into one of two groups: normal PC level or elevated PC level (according to the respective cutoff values). All major features of the included studies are presented in Table 1. We performed meta-analyses of the three groups separately in order to avoid the influence of heterogeneity and to obtain detailed results.

**Table 1:** Major feature of the included studies.

Study	Country	Study design	Case number	Cut-off/nL	Median age, y	Median follow-up, mo	Survival analysis
*All-Stage Group*							
Jacoben_2002	Sweden	R	161	306	66(25–85)	D:15(1–131) A:126(51–206)	OS
Inoue_2004	Japan	R	196	400	60.4^a^	62.6^a^ (1–202)	CSS
Bensalah_2006	France	R	804	450	62	50(10–265)	CSS
Lee_2006	Korea	R	375	354	55(26–81)	D:24.5(3.5–100.5) A:55.5(1–140.5)	CSS,PFS
Karakiewicz_2007	Canada, US	R	1828	450	63(10–94)	25.2(1.2–273.6)	CSS
Pflanz_2008	Germany	R	607	400	63.1(18–84)	54	OS,CSS
Brookman-Amissah_2009	Germany	R	771	400	62.2(18–84)	67	OS,CSS
Brookman-May_2013	Germany	R	3139	400	63.6(55.5–70.4)	69.5(35–105)	CSS
Fox_2013	Australia	P	362	400	62(19–84)	-	OS
Yap_2013	Australia	R	151	400	60.7^a^ (34–83)	26(0.2–193.6)	CSS
Yang_2013	China	R	366	400	-	29.1	OS
*Localized Group*							
Gogus_2004	Turkey	R	152	400	58.4^a^ (30–78)	34(3–91)	CSS
Lehmann_2004	Germany	R	48	338.5	63(35–82)	D:8(5–16) A:91(75–120)	CSS,RFS
Erdemir_2007	Turkey	R	118	400	61.4^a^ (30–78)	52.7^a^ (9–96)	CSS
Ramsey_2008	UK	P	83	400	-	38(19-)	CSS
Dae_2011	Korea	R	177	M:380 F:370	53.5^a^ (28–83)	48(13–111)	RFS
*Metastatic Group*							
Suppiah_2006	US, France	R	700	400	-	28.8	OS
Cho_2008	Korea	R	197	450	55.1^a^ (24–83)	22.6^a^ (3–120)	CSS
Heng_2009	Canada, US	R	645	-	60(13–88)	24.5	OS
Richey_2011	US	R	188	-	60.8(18.2–83.9)	D:6.9 A:13.1(1–64.6)	OS
Beuselinck_2013	Europe	R	88	400	59^a^ (38–84)	46(1–73)	OS,PFS
Dirican_2013	Turkey	R	58	312.5	61(40–79)	34(5–58)	OS
Yildiz_2013	Turkey	P	77	450	58(26–80)	18.5(2–43)	OS,PFS
Park_2014	Korea	R	83	-	57(33–80)	18(1–62)	OS,PFS
Stenman_2014	Sweden	R	84	360	67(31–93)	20	OS

Study design is described as prospective (P) or retrospective (R). OS overall survival, CSS cancer-specific survival, PFS progression-free survival, RFS relapse-free survival.

^a^ Reported as mean age or mean follow-up time.

We initially focused on the differences in the survival of patients with normal PC level versus elevated PC level. The hazard ratios (HRs) of OS, CSS, PFS, and RFS were combined for analysis. In these meta-analyses, univariate HRs and 95% CIs were calculated. Some of the studies provided HR and 95% CI explicitly. If the HR and 95% CI were not available, we calculated the values using the original study data by using the methods reported by Tierney et al. [34].

We then studied the relationships between an elevated PC level and the clinicopathological features of RCC. Data on TNM stage (III/IV versus I/II), pathological T stage (T3/T4 versus T1/T2), lymph node metastasis (positive versus negative), distant metastasis (positive versus negative), Fuhrman grade (III/IV versus I/II), tumor size (>7 versus < = 7), and Eastern Cooperative Oncology Group (ECOG) score (>0 versus 0) were dichotomized. The odds ratio (OR) and corresponding 95% CI were extracted and used in meta-analysis.

A key point of our study was that the cutoff level varied and ranged from 306 to 450 (cells/nL), which was attributed to the use of different methods and kits for measuring platelet count in respective clinical centers. As most of the studies did not indicate detailed information regarding the methods and kits for measuring platelet count level, it was extremely puzzling for us to stratify the cases (normal or elevated platelet count) and merged the data. A feasible way was just to classify all the cases according to their original studies (normal or elevated platelet count). Naturally, it would develop heterogeneity and bias, together with patients’ baseline characteristics (race, country, gender, age, etc.).

### Statistical analysis

A test of heterogeneity of combined HRs and ORs was conducted using Cochran’s Q test and Higgins *I*-squared statistic. A *P* value of less than 0.1 was considered significant. *I* ^2^ values of >50% indicated heterogeneity among studies. When heterogeneity was significant, we used a random-effect model. Otherwise, we used a fixed-effect model. For the analysis of the association of PC level with clinicopathological characteristics, ORs and their 95% CIs were determined. An observed HR or OR > 1 implied poor survival for the group with an elevated PC level or a significant association between an elevated PC level and clinicopathological features. We pooled HRs and ORs of the studies by using Stata 12.0 software (StatCorp, College Station, TX, USA). For the analysis of heterogeneous studies, we also conducted sensitivity analysis by omission of each single study to evaluate stability of the results. Publication bias was evaluated by funnel plot visual inspection for all of the comparisons and by using Begg (rank correlation analysis) and Egger tests (linear regression analysis) where applicable (number of included studies > 6).

## Results

A total of 892 records were retrieved from the primary literature search (of the aforementioned databases), and 2 reports were identified for inclusion by searching the references. Of these, 131 duplicates were excluded. After screening the titles, abstracts, publication types, and full text of the remaining records, 27 articles qualified for the present analysis. However, 2 articles showing only the Kaplan-Meier curves without providing essential data were subsequently excluded. Finally, 25 studies, with a total of 11,458 patients, met our eligibility criteria for this systematic review (Table 1).

At first, we evaluated survival at different PC levels. To avoid the influence of heterogeneity and obtain detailed results, we performed meta-analysis of the different groups separately. Because the all-stage group had only one analysis of PFS, and the metastatic group had only one of CSS, we did not include these categories in their respective groups. However, the study reported by Brookman-May et al. [16], which was classified in the all-stage group, also presented the results of CSS in localized RCC, and these data were therefore included in the localized RCC group.

**Fig 2:**
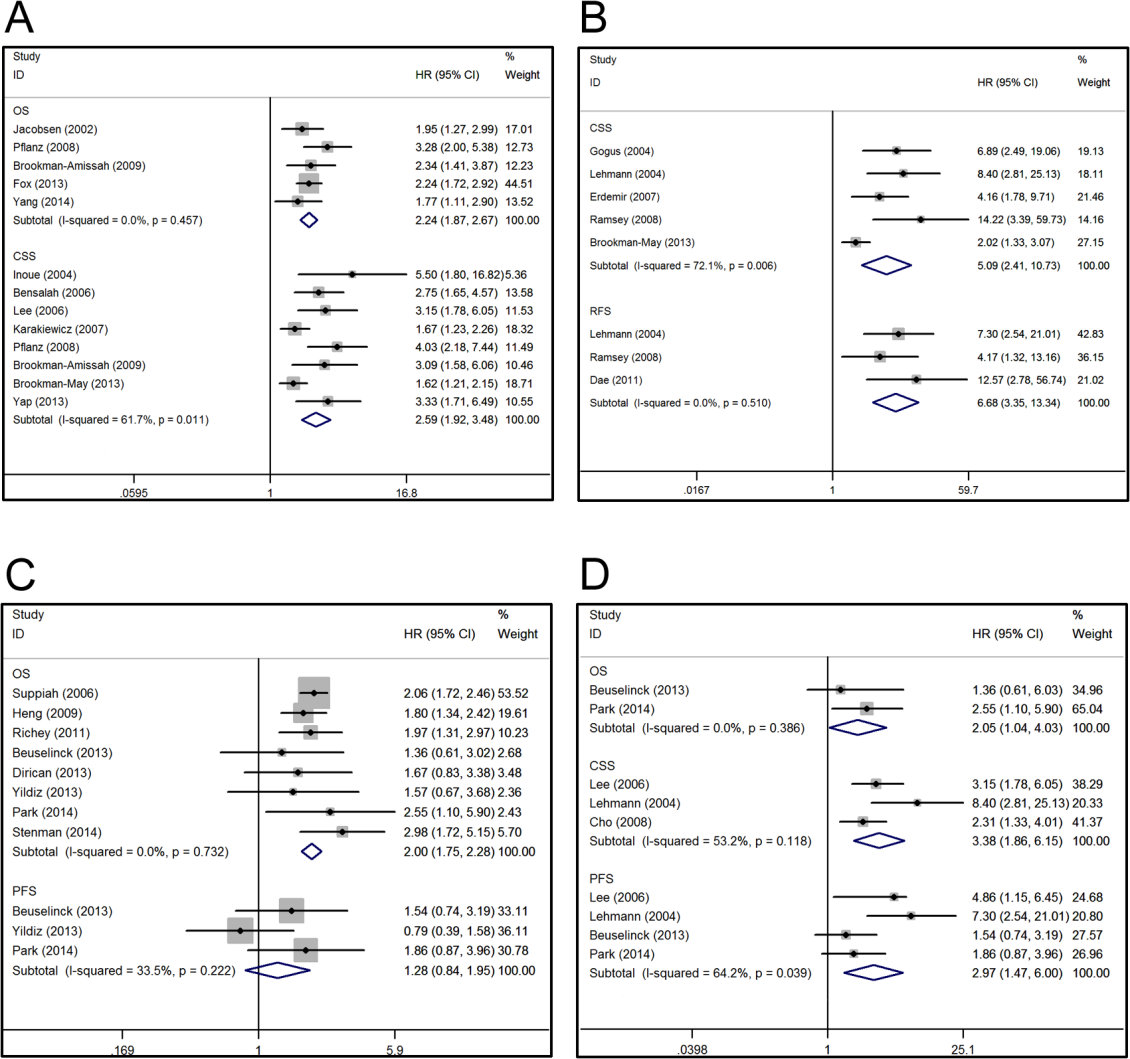
Forest plots of studies evaluating hazard ratios of elevated PC level compared with normal level. (A) Elevated PC level was associated with shorter overall survival and cancer-specific survival in renal cell carcinoma (All-stage group); (B) Elevated PC level was associated with poorer cancer-specific survival and recurrence-free survival in localized RCC (Localized group); (C) For metastatic RCC, elevated PC level was associated with worse overall survival but not progression-free survival (metastatic group); (D) For clear cell RCC, elevated PC level was associated with poorer overall survival, cancer-specific survival and progression-free survival.

In the all-stage group, elevated PC level was associated with shorter OS (HR 2.24, 95% CI 1.87–2.67, *P*<0.001; a fixed-effect model was used in the absence of evidence for inter-study heterogeneity) and CSS (HR 2.59, 95% CI 1.92–3.48, *P*<0.001; a random-effect model was used because of the heterogeneity [*P* = 0.011]) (Fig 2A). Additionally, Lee et al. [4] reported poorer PFS in patients with thrombocytosis (HR 4.86, 95% CI 1.15–6.45, *P*<0.001). In the localized group, an elevated PC level was associated with poorer CSS (HR 5.09, 95% CI 2.41–10.73, *P*<0.001) and RFS (HR 6.68, 95% CI 3.35–13.34, *P*<0.001) (Fig 2B). Because of the inter-study heterogeneity in some cases and not others, either a random-effect model or a fixed-effect model was applied in the respective analyses. For instance, a random-effect model was applied in the analysis of CSS, while a fixed-effect model was applied for RFS. None of the studies in the localized group reported data for OS or PFS. In the metastatic group, an elevated PC level was associated with worse OS (HR 2.00, 95% CI 1.75–2.28, *P*<0.001) (Fig 2C). However, the association between an elevated PC level and PFS (HR 1.28, 95% CI 0.84–1.95, *P* = 0.246) was not significant (Fig 2C). As no heterogeneity was observed, we used a fixed-effect model. Additionally, Cho et al. [26] reported poorer CSS in patients with elevated PC (HR 2.31, 95% CI 1.33–4.01, *P* = 0.003) in metastatic RCC.

Five studies focused on clear cell RCC (ccRCC)—one study was classified in the all-stage group, one in the localized group, and three in the metastatic group. We combined these data and evaluated the survival of patients with different PC levels and ccRCC. We found elevated PC levels to be associated with poorer OS (HR 2.05, 95% CI 1.04–4.03, *P* = 0.038), CSS (HR 3.38, 95% CI 1.86–6.15, *P*<0.001) and PFS (HR 2.97, 95% CI 1.47–6.00, *P* = 0.002; random-effect model for heterogeneity) (Fig 2D). For the association between elevated PC levels and clinicopathological features, we performed an overall analysis of patients in all three groups. As shown in Table 2, an elevated PC level had a significant association with TNM stage (III/IV versus I/II: OR 3.11, 95% CI 1.59–6.06, *P* = 0.001), pathological T stage (T3/T4 versus T1/T2: OR 3.13, 95% CI 2.60–3.77, *P*<0.001), lymph node metastasis (positive versus negative: OR 4.01, 95% CI 2.99–5.37, *P*<0.001), distant metastasis (positive versus negative: OR 3.85, 95% CI 2.46–6.04, *P*<0.001), Fuhrman grade (III/IV versus I/II: OR 3.70, 95% CI 3.00–4.56, *P*<0.001), tumor size (>7 versus < = 7: OR 4.69, 95% CI 2.78–7.91, *P*<0.001), and ECOG score (>0 versus 0: OR 5.50, 95% CI 3.26–9.28, *P*<0.001). We found some inter-study heterogeneity (*I* ^2^ = 60%) in the distant metastasis, whereas the analysis of other clinicopathological parameters did not exhibit heterogeneity (*I* ^2^ 0–9.6%).

**Table 2:** Meta-analysis of the association between elevated PC and clinicopathological features of renal cell carcinoma.

Variables	Studies	Patients	Pooled OR	95% CI	*P* value	Heterogeneity *I* ^2^ (%)	*P* value
**TNM stage**	2	319	3.11	1.59–6.06	0.001	0	0.631
**pT stage**	8	5666	3.13	2.60–3.77	<0.001	0	0.457
**Lymph node metastasis**	6	4552	4.01	2.99–5.37	<0.001	9.6	0.354
**Distant metastasis**	5	4906	3.85	2.46–6.04	<0.001	60	0.039
**Fuhrman grade**	7	4708	3.70	3.00–4.56	<0.001	0	0.641
**Tumor size**	3	1122	4.69	2.78–7.91	<0.001	0	0.791
**ECOG score**	2	922	5.50	3.26–9.28	<0.001	0	0.412

**Fig 3:**
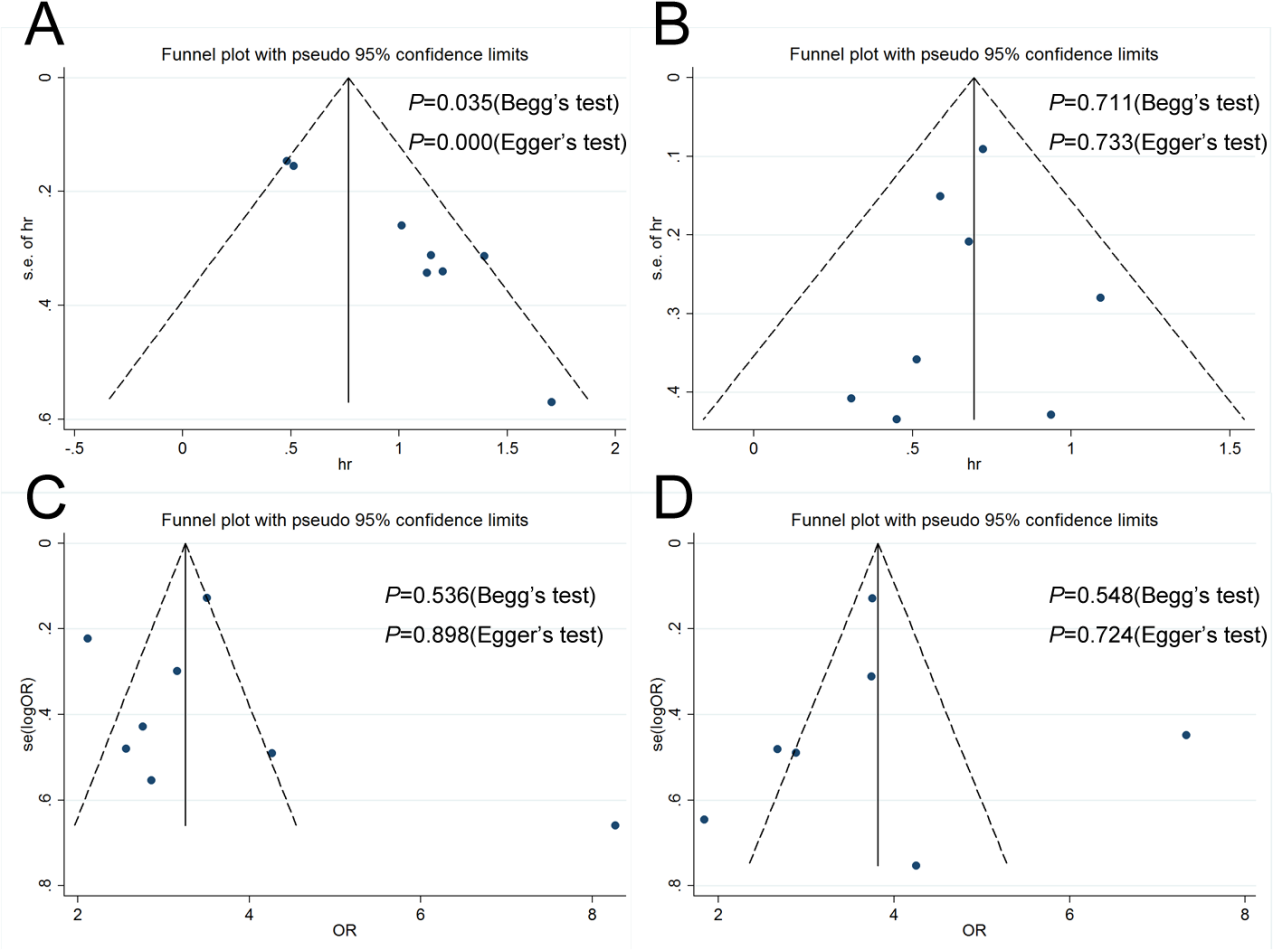
Funnel plots, Begg and Egger tests result for the evaluation of potential publication bias. Plots are arranged as follows: (A) RCC CSS; (B) MRCC OS; (C) PC pT stage; (D) PC Fuhrman grade.

Finally, publication bias of the included studies was evaluated by using funnel plots and the Begg and Egger tests. As presented in Fig 3, most of the funnel plots are symmetric. Of the four meta-analyses, three indicated no evidence of significant publication bias. A publication bias was identified in only the RCC CSS studies based on Begg (*P* = 0.035) and Egger (*P* = 0.000) tests (Fig 3A). Sensitivity analysis indicated that omitting any single study did not significantly affect the pooled HR or OR.

## Discussion

In 1964, Levin and Conley [35] initially described the association of thrombocytosis with cancer. They found that 38% of adult inpatients with thrombocytosis had unrecognized primary cancers. Thrombocytosis has since been identified as a prognostic factor for many types of cancer [5,6], and the PC level, which reflects the systemic inflammatory response, and has been associated with adverse implications in the prognosis of RCC. However, this link is still controversial, possibly due to the lack of reliable, clear-cut results that do not require further confirmatory research; this situation implies a need for a systematic review and meta-analysis of the current literature.

In the current study, we performed separate meta-analysis of different groups. In line with a previous study [7], we identified that an elevated PC level predicts poor survival in patients with RCC; thus, patients suffering from thrombocytosis in RCC are likely to have shorter OS and CSS. In subgroup analysis of localized RCC, patients with thrombocytosis had worse CSS and RFS. Among patients with metastatic RCC, those with normal-range PC levels had more promising outcomes although the PC level showed a limited predictive effect for PFS. Finally, for patients with clear cell RCC, an elevated PC level predicted poor OS, which was also true for CSS and PFS.

Our results also indicate that RCC patients with elevated PC levels are likely to have a higher TNM stage, a higher pathological T stage, positive lymph nodes, distant metastasis, a higher Fuhrman grade, a larger tumor size, and a higher ECOG score than RCC patients with normal PC levels, which may imply that PC is not an independent prognostic marker for RCC. Even so, our results show that PC is strong predictor of prognosis in RCC. As platelet count measurement is well standardized and available in every clinical laboratory, it could be a helpful and convenient serum biomarker for clinical practice.

The reasons why thrombocytosis might be of prognostic relevance in patients suffering RCC as well as other solid malignancies remain speculative at this time. Some evidence suggests that platelets protect circulating tumor cells from detection or attack by the immune system by facilitating cancer cell adhesion to the vascular endothelium through the formation of tumor thrombi, and by interacting with tumor cells through platelet ligands [27]. Platelets may also promote tumor cell growth by secreting several angiogenic and tumor growth factors, such as thrombospondins, endostatin, vascular endothelial growth factor (VEGF), platelet-derived growth factor (PDGF), and hepatocyte growth factor [36]. Moreover, thrombocytosis could also result from the host’s response to growth factors secreted by the tumor, which may directly increase the metastatic risk and worsen prognosis [37]. However, if thrombocytosis is the primary event, the specific mechanism for its development is unclear. Ito et al. [38] proposed that thrombocytosis and other factors (such as a higher C-reactive protein) lead to increased interleukin-6 levels, which activate T-lymphocytes and macrophages, causing a prolonged T helper type 2 cytokine response [23,39]. This process might play an important role in the progression of RCC.

Several limitations of this study need to be acknowledged. First, although 25 studies of 11,458 patients were used in this meta-analysis, they were categorized into three independent groups and analyzed separately. This avoided the influence of heterogeneity and retrieved detailed results (as intended), but simultaneously decreased the data for special subgroups. Second, marked heterogeneity of subjects was seen in some analyses. The heterogeneity of the population was probably due to differences in factors such as study design, patients’ baseline characteristics (race, country, gender, age, and tumor stage and grade), cut-off values for PC, patients’ treatment, and duration of follow-up. In addition, the method of calculating the HRs of these studies was also a potential factor that might have led to heterogeneity. Of the 25 studies, 13 directly provided HRs, and individual HRs of the remaining studies were calculated using the methods reported by Tierney et al. [34]. The calculated HRs could be not as dependable as those retrieved directly from reported statistics. In analyzing associations between PC and clinicopathological features of RCC, as well as that between PC and survival in ccRCC, all three groups had studies that lacked complete data, which may have contributed to the heterogeneity and rendered the results less reliable. Furthermore, a publication bias was detected in the analysis of CSS in RCC, which cannot be properly overcome by statistical techniques. Finally, meta-analysis is now a widely used technique for summarizing evidence from multiple studies. It is particularly useful when there is uncertainty about the potential benefit and/or harm of an intervention and when there are practice variations. Nevertheless, the unavoidable limitations exist. All meta-analyses are affected by the quality of their component studies; the fact that research with statistically significant results is potentially more likely to be submitted and published than work with null or non-significant results, could compromise the validity of such analyses [40]. Furthermore, the current meta-analysis of published studies does not have the benefit of currently unpublished data [41]. Owing to further groundbreaking research on inflammation and tumors, we believe that the use of PC as a prognostic marker for RCC will be extensively studied, and additional studies supporting our results will facilitate a consensus on this matter.

## Conclusion

Our comprehensive meta-analysis strongly supports an inverse relationship between PC and prognosis in RCC. The relative availability and low cost of this biomarker should facilitate its use in this context, although a large prospective study is needed to confirm our findings.

## Supporting Information

S1 PRISMA Checklist PRISMA checklist. (DOC)
